# Retinochoroidal Vascular Changes in Long-Term Type 1 Diabetic Patients Assessed by Optic Coherence Tomography Angiography

**DOI:** 10.3390/biomedicines12081780

**Published:** 2024-08-06

**Authors:** Maria Sopeña-Pinilla, Elvira Orduna-Hospital, Maria D. Diaz-Barreda, Ana Boned-Murillo, Guisela Fernandez-Espinosa, Marta Arias-Alvarez, Javier Acha-Perez, Ana Sanchez-Cano, Isabel Pinilla

**Affiliations:** 1 Department of Ophthalmology, Miguel Servet University Hospital, 50009 Zaragoza, Spain; mariasopenapinilla@gmail.com; 2 Aragón Health Research Institute (IIS Aragón), 50009 Zaragoza, Spain; eordunahospital@unizar.es (E.O.-H.); lodiba92@gmail.com (M.D.D.-B.); anabomu@hotmail.com (A.B.-M.); guisela.fernandez3@gmail.com (G.F.-E.); martariasalvarez7@gmail.com (M.A.-A.); j.acha.perez@gmail.com (J.A.-P.); anaisa@unizar.es (A.S.-C.); 3 Department of Applied Physics, University of Zaragoza, 50009 Zaragoza, Spain; 4 Department of Ophthalmology, Hospital Obispo Polanco, 44002 Teruel, Spain; 5 Department of Neurophysiology, Lozano Blesa University Hospital, 50009 Zaragoza, Spain; 6 Department of Endocrinology, Miguel Servet University Hospital, 50009 Zaragoza, Spain; 7 Department of Ophthalmology, Lozano Blesa University Hospital, 50009 Zaragoza, Spain

**Keywords:** diabetes mellitus, diabetic retinopathy, foveal avascular zone, retinal vascularization, optical coherence tomography angiography

## Abstract

To study retinal and choriocapillaris (CC) alterations using optical coherence tomography angiography (OCTA) in long-term type 1 diabetic (DM1) patients without diabetic retinopathy (DR). Seventy-eight eyes from 78 well-controlled DM1 patients diagnosed at least 15 years prior and 130 eyes of 130 healthy subjects were included in a cross-sectional descriptive study. Six eyes were excluded from the DM1 group. OCTA with Deep Range Imaging (DRI)-Triton swept source (SS)-OCT was performed. Statistically significant differences were found in all areas of the superficial capillary plexus (SCP), with lower values in DM1 patients. Differences were noted in all quadrants of the deep capillary plexus (DCP) except for the central area. Significant changes in CC blood flow were only found in the center. The foveal avascular zone (FAZ) area and diameters in the SCP were significantly different, while the DCP FAZ area was similar in both groups. Disease duration and microalbuminuria correlated negatively with some SCP areas and positively with FAZ values. Anatomical evaluation revealed microaneurysms in both plexuses, FAZ modifications, and areas lacking blood perfusion. Long-term type 1 diabetic patients without DR display microvascular abnormalities affecting retinal and CC blood perfusion, along with anatomical changes in retinal blood vessels.

## 1. Introduction

Diabetes mellitus (DM) is among the most prevalent metabolic disorders worldwide, and its incidence is steadily rising, with an estimated 51% increase in its prevalence projected by 2045 [1]. Type 1 DM (DM1) represents 5–10% of DM cases and is caused by the autoimmune destruction of pancreatic beta cells [2]. Following the criteria established by the American Diabetes Association (ADA), DM may be diagnosed based on plasma glucose criteria or glycated hemoglobin (HbA1c) criteria: fasting plasma glucose ≥ 126 mg/dL, or 2 h plasma glucose ≥ 200 mg/dL during an oral glucose tolerance test, or HbA1c ≥ 6.5%, or random plasma glucose ≥ 200 mg/dL in patients with classic symptoms of hyperglycemia or hyperglycemic crisis. In the absence of unequivocal hyperglycemia, diagnosis requires two abnormal test results from the same sample or in two separate test samples [3]. The onset of chronic complications in DM1 depends on the disease progression and management. However, some patients with prolonged disease duration do not develop complications, possibly due to other factors such as residual beta cell secretion, detectable by ultrasensitive C-peptide [4]. Additional factors contributing to microangiopathy-related complications such as diabetic retinopathy (DR) include glycemic variability, coexistence of other autoimmune diseases, insulin resistance (eGDR), etc. [5].

DR is recognized as a highly specific neurovascular complication of DM [6]. Diabetic macular edema and complications related to proliferative DR are the main cause of visual loss in these patients. The time span between the onset of DM and the appearance of DR can vary. Prior to its establishment, changes in the inner retina characterized by cell apoptosis and reductions in both ganglion cells and the retinal nerve fiber layer have been documented [7,8,9]. Neuronal damage and microvascular changes that are undetectable by standard ophthalmic explorations occur prior to the observable signs of DR [10]. Early detection of these subclinical changes is crucial to impede disease progression. These changes can be identified by optical coherence tomography angiography (OCTA). OCTA is a non-invasive diagnostic tool that can detect changes in the retinal and choroidal capillary plexuses [11]. OCTA permits visualization of alterations in the retinal capillary plexuses, including the superficial capillary plexus (SCP), intermediate capillary plexus (ICP), deep capillary plexus (DCP), radial peripapillary plexus, and the choriocapillaris (CC) flow [12]. Capillary dropout or modifications in the foveal avascular area (FAZ) could impact the demands of highly metabolically active retinal neurons, leading to neural degeneration and impairment of visual acuity (VA). The presence of such changes could serve as biomarkers for disease progression.

The aim of this study was to evaluate and compare flow and anatomical alterations in the retinochoroidal capillary plexuses using OCTA between long-term DM1 patients without DR and healthy subjects. We sought to analyze the perifoveal capillary network and assess changes in the size and morphology of the FAZ and to investigate potential correlations between OCT findings and metabolic and ocular parameters. Although other studies have provided similar data, we have only studied DM1 patients with a disease progression of over 15 years. It is uncommon to find studies focused on such long-term patients.

## 2. Materials and Methods

We performed a cross-sectional study including patients with DM1 and healthy control subjects recruited from the Ophthalmology Department of the University Hospital in Zaragoza, Spain. One eye of each participant was randomly selected. This study was conducted between January 2021 and June 2023.

Ethical approval was obtained from the Ethics Committee of Clinical Investigation and the Aragon Clinical Research Committee with identification numbers PI17/0298 and PI23/063. This study adhered to the principles of the Helsinki Declaration and complies with Spanish legislation in the field of biomedical research and the protection of personal data (Organic Law 3/2018 and Laws 41/2002 and 14/2007 on biomedical research). Informed consent was obtained from both patient and control group members.

All DM1 patients were under the care of the endocrinology unit and demonstrated good glycemic control, as indicated by levels of HbA1c, lipid profile, and arterial blood pressure within tight limits. 

Inclusion criteria for the DM1 group were the following: a confirmed diagnosis of DM1 at least 15 years prior, aged between 18 and 65 years, Caucasian ethnicity, and provided written informed consent; best corrected VA (BCVA) ≥ 20/25 on Snellen charts, with a spherical equivalent under ±5D or astigmatism <3D, and a clear lens or lens opacities graded lower than 1.0 in the Lens Opacities Classification System III (LOCS III). Exclusion criteria included any signs of DR on fundus examination or wide-field retinography, intraocular pressure (IOP) over 20 mmHg, or any optic disc changes suggestive of glaucoma. Additionally, a history of any ocular disease, intraocular surgery, and uncontrolled systemic pathologies, such as cardiovascular disease, were grounds for exclusion. The control group were age-matched with the DM1 group. Inclusion criteria were the same as for the DM1 group, except for the presence of DM. Exclusion criteria added the history of DM disease.

All patients underwent a comprehensive ophthalmic examination, which included assessment of BCVA converted to the logarithm of the minimal angle of resolution (LogMAR), measurement of axial length (AL) via optical biometry Aladdin KR-1W (Topcon Eye Care Company, Tokyo, Japan), slit lamp examination of the anterior and posterior segments, Goldmann tonometry, indirect ophthalmoscopy, wide-field retinography with Clarus 700 (Carl Zeiss Meditec, Dublin, OH, USA) swept-source OCT (SS-OCT), and acquisition of 3 × 3 mm OCTA images using a DRI-Triton SS-OCT (Topcon Eye Care Company, Tokyo, Japan) with IMAGEnet 6 software Version 1.22.1.14101© 2014. Fundus examination and results of both OCT and Clarus acquisitions were evaluated by 2 retina experts to ensure the absence of DR lesions. OCTA was performed without pupil dilation. Vessel density (VD) measurements were obtained for SCP, DCP, and CC within a 3 mm diameter grid divided into 5 areas: a central area of 1 mm in diameter, surrounded by 4 parafoveal quadrants (superior (S), temporal (T), inferior (I), and nasal (N)). VD was expressed as the % of total area occupied by blood vessels (% of positive pixels vs. total pixels in area of interest), with calculations performed automatically after adjusting the references in the device. 

Manual measurements of the FAZ area and both horizontal and vertical diameters in the SCP and DCP were conducted using the measuring tool provided by the device, and the average of the readings of two independent examiners was considered (I.P., M.S-P.). DRI-Triton SS-OCT does not distinguish between DCP and ICP. VD was assessed for all 5 areas in the SCP, the DCP, and the CC, while FAZ metrics were evaluated for both the SCP and the DCP. Morphological changes in OCTA images were analyzed to identify any microvascular abnormalities, with agreement reached between two independent examiners (IP, MSP). These changes included FAZ modifications in DCP and SCP, microaneurysms (MAs), and localized areas of capillary dropout; when capillary dropout was considerable, the images were described as ischemic. All included images were adequately centered and of high quality, with a signal strength exceeding 60 out of a total of 100.

Regarding the statistical analysis, all data were collected using Microsoft Excel (Microsoft Excel for Mac. Version 16.71. Microsoft Corp. Redmond, WA, USA). Statistical analyses were conducted using the Statistical Package for the Social Sciences (SPSS 25.0, SPSS, IBM, Armonk, NY, USA). Results were presented as mean and standard deviation (SD) for quantitative variables and as number of cases and percentages for qualitative data. Prior to analyzing quantitative variables, normal distribution was assessed using the Kolmogorov–Smirnov test. Since values were found to follow a normal distribution, parametric tests were employed for the analysis (*t*-test for independent samples). The Pearson correlation test was used to explore correlation between variables. A *p*-value < 0.05 was considered statistically significant.

## 3. Results

The DM1 group comprised 84 eyes of 84 patients. After analyzing image quality, six eyes were excluded, resulting in a final sample of 78 eyes. Forty participants were male (57.2%) and thirty-eight were female (42.8%). Regarding eye distribution, 41 eyes were right eyes (REs) and 37 were left eyes (LEs) (58.6 and 41.4%, respectively). The control group consisted of 130 eyes from 130 subjects. Sixty-two participants were male (47.7%) and sixty-eight were female (52.3%); 68 eyes were REs and 62 were LE (52.3% and 47.7%, respectively). Disease duration and biochemical data are presented in Table 1. No significant differences were observed in age, sex, VA, SE, or AL between both groups (Table 2).

**Figure 1:**
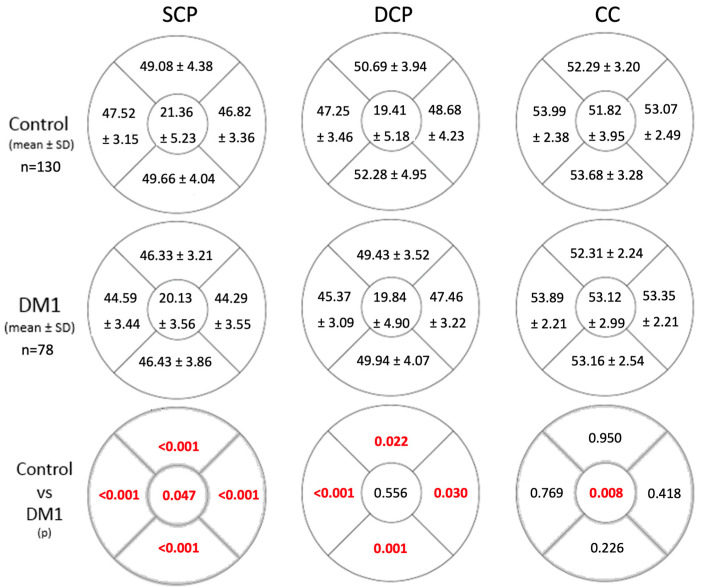
Flow values in the superficial capillary plexus (SCP), deep capillary plexus (DCP), and choriocapillaris (CC) in the control group and the DM1 group. Statistically significant differences between flow values of both groups are indicated in the lower grid row in red (*p* < 0.05). Abbreviations: SCP, superficial capillary plexus; DM1, type 1 diabetes mellitus; *p*, statistical significance level. Blood flow is measured as the % of pixels with a positive sign relative to the total pixels in the studied area. The figures represent a right eye.

Flow in the SCP was significantly reduced across all the studied areas in the DM1 group (Figure 1). There were differences in the FAZ area as well as both diameters between groups, with higher values in the diabetic group (Table 3). Flow in the DCP exhibited statistically significant differences in all quadrants except the central zone (Figure 1). However, FAZ area and diameters in the DCP were similar between both groups (Table 3). In the CC, flow differences were only observed in the central area. DM1 patients exhibited higher flow compared to the control group (Figure 1).

**Figure 2:**
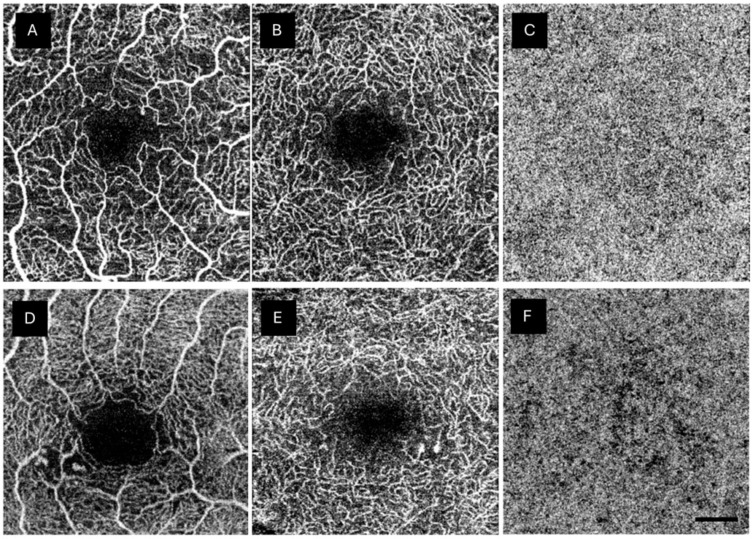
Anatomical changes shown in the type 1 diabetes mellitus (DM1) group by optical coherence tomography angiography (OCTA). (**A**) shows abnormalities in the foveal avascular zone. (**A**,**B**,**D**) depict capillary drop out. (**D**,**E**) display microaneurysms in both SCP and DCP, respectively, surrounded by areas with capillary loss. (**C**) shows a normal choriocapillaris plexus (CC), while (**F**) reveals a lack of capillaries in the CC. The scale bar (lower right corner) represents 500 microns.

Anatomical abnormalities were investigated in both retinal plexuses and the CC of the DM1 group. The most frequent abnormality was the presence of MAs in the DCP, affecting 56.8% of the studied eyes. MAs were also found in the SCP (47.5%). Three patients showed large MAs in both retinal plexuses. Various degrees of ischemia were detected in 88.4% and 78.2% of DM1 patients in the SCP and DCP, respectively. Some degree of reduced CC perfusion was observed in 66.6% of the patients (Table 4). OCTA findings were considered normal in 8.9%, 10.2%, and 33.3% in the SCP, DCP, and CC, respectively. Figure 2 shows an example of the anatomical abnormalities observed in the DM1 group.

We examined correlations between BCVA (measured with Snellen), age, duration of the disease, microalbuminuria, and HbA1c levels with the flow in each plexus and FAZ parameters. No significant correlations were observed between BCVA and the SCP. However, significant correlations were found between BCVA and the DCP with the C area (r = 0.282, *p* = 0.032) and the horizontal FAZ diameter (r = −0.262, *p* = 0.045). This suggests that a larger horizontal FAZ diameter and lower DCP flow in the foveal area (C) are associated with poorer BCVA. A negative correlation was observed between CC flow in the C area and BCVA (r = −0.298, *p* = 0.38). Age demonstrated significant positive correlations with urea, creatinine, and HbA1c levels (r = 0.359, *p* = 0.003; r = 0.258, *p* = 0.023; r = 0.269, *p* = 0.017, respectively). Negative correlations were found between age and CC flow in the C and T areas (r = −0.273 *p* = 0.016, r = −0.240 *p* = 0.034, respectively), while a positive correlation was observed with the N area of the SCP (r = 0.350, *p* = 0.002).

The duration of the disease showed significant negative correlations with three SCP areas (S: r = −0.266, *p* = 0.019; T: r = −0.422, *p* < 0.001; and I: r = −0.251, *p* = 0.028). With a longer duration of the disease, the FAZ of the SCP tends to enlarge both in size and horizontal diameter (FAZ area: r = 0.277, *p* = 0.015; FAZ horizontal diameter: r = 0.261, *p* = 0.023). Microalbuminuria exhibited significant negative correlations with certain SCP areas (C: r = −0.292, *p* = 0.011 and S: r = −0.242, *p* = 0.034) and with the S area of the DCP (r = −0.261, *p* = 0.023). Positive correlations were observed between microalbuminuria and FAZ area for both plexuses (SCP FAZ area: r = 0.321, *p* = 0.005 and DCP FAZ area: r = 0.230, *p* = 0.047), as well as with the SCP FAZ diameters (FAZ horizontal diameter: r = 0.252, *p* = 0.029 and FAZ vertical diameter: r = 0.252, *p* = 0.029). Microalbuminuria was also positively correlated with HbA1c levels (r = 0.331, *p* = 0.003). However, no correlations between HbA1c levels and the flows of the different capillary plexuses were found. Lastly, significant positive correlations were observed between the C area of both SCP and DCP (r = 0.684, *p* < 0.001). Negative correlations were found between both SCP and DCP C areas and various FAZ parameters of both plexuses. No correlations were observed with the CC (Table 5).

## 4. Discussion

Our study reveals preclinical changes assessed by OCTA in long-term DM1 patients without DR. Detecting these changes prior to the onset of the first vascular signs of DR is crucial in order to identify patients at higher risk of developing retinal manifestations. Using several imaging techniques such as wide-field OCTA imaging and other metabolomic or biochemical markers could enable us to identify alterations in patients without evident DR signs [13], expanding our therapeutic options to include neuroprotective, anti-inflammatory, or antioxidant agents.

Our patients had a prolonged duration of the disease (mean duration 26.56 years) with a mean HbA1c of 7.46%. The absence of complications in these patients has been attributed to the persistence of superactive C-peptide, among other unknown factors [4,14]. However, protective factors alone cannot account for the varying degrees of impairment observed in the retina, kidneys, or cardiovascular system. It is likely that there are distinct factors associated with each complication. Although these patients had no signs of DR, we did find varying degrees of ischemia in a high number of our patients, with 88.4% and 78.2% showing signs of ischemia in the SCP and the DCP, respectively. Additionally, approximately half of the subjects exhibited MAs mainly in the DCP. These findings suggest that vascular changes are present in these patients even in the absence of clear signs of DR.

### 4.1. FAZ Morphology and Metrics Modifications

We were unable to identify differences in FAZ area between the DM1 and control groups in either of the studied retinal plexuses using OCTA. FAZ modifications have been described in patients both with and without DR signs. A meta-analysis by Zhang et al. found a larger FAZ in DM patients without DR compared to controls [15]. De Carlo et al. were the first authors to assess OCTA changes in patients without DR lesions [16]. They reported an enlargement of the FAZ in DM1 and DM2 patients, along with morphological abnormalities using full-thickness images of the retinal vasculature. The FAZ area values were slightly higher than in our series, using the angioVue system. They also described vascular morphological changes. Takase et al. also found FAZ enlargement in DM1 patients even if they did not have DR [17]. Their study revealed an increase in FAZ size parallel to the severity of DR. The FAZ areas reported by Di et al., based on full-thickness vasculature images, were also found to be increased in DM patients without DR, and these areas increased further with the extent of retinal damage [18]. 

### 4.2. Changes in Vascular Density

Our study reveals that long-standing DM1 patients exhibit decreased VD in both the SCP and DCP in the parafoveal areas, with no alterations observed in the C area of either plexus. We observed an increase in flow in the C area of the DM1 CC. The CC flow was found to be negatively correlated with the BCVA. This increase in photoreceptor blood supply could potentially be related to a higher level of oxidative stress and subsequent cell damage.

Similar findings were reported by Cao et al. [19] in DM2 patients with shorter disease duration (< 5 years): a diminished VD in both plexuses and the CC with no differences observed in FAZ areas. They reported MAs or perfusion deficits in only 15 and 25% of cases, respectively. Authors like Dimitrova et al. [20] have also reported changes in VD in both plexuses, while others like Simonett et al. [21] and Carnevali et al. [22] reported changes only in the DCP. However, some of the inconsistencies found upon evaluating the DCP could be attributed to limitations in OCTA technology and the presence of projection artifacts [23]. Differences between DM1 and DM2 patients have also been described [24]. 

We have only evaluated the 3 × 3 mm OCTA scan, known for its higher reproducibility compared to other OCTA grids like 6 × 6 mm. A reduction in VD in both SCP and DCP has been revealed in the peripheral retina using ultrawide OCTA, although average VD in the central macula has proven to be more predictive of DR development, particularly in the SCP [25]. Moreover, angiocube 3 × 3 mm scans acquired with DRI-Triton OCT offer higher resolution. Other OCTA systems, such as the PLEX Elite 6 × 6, have been described to detect earlier vascular changes [26]. 

### 4.3. Correlation Study

In our correlation study, we did not observe any relationships with HbA1c levels. However, other authors have reported associations between OCTA metrics, HbA1c levels, and FAZ circularity in DM1 patients without DR [27]. We found that patients with longer disease duration exhibited decreased flow in the SCP in both vertical areas and the temporal region. Additionally, we observed a correlation between FAZ parameters and the duration of the disease. Longer durations of DM1 were associated with larger FAZ areas and horizontal diameters. 

### 4.4. Morphological Changes

The ability of OCTA to visualize MAs is well established [28]. While OCTA may detect fewer MAs compared to fluorescein angiography, it can localize them within the retinal plexuses. Salz et al. [29] demonstrated that OCTA has a sensitivity and specificity of 85% and 75%, respectively, for MA detection compared to fluorescein angiography. In our study, we identified 50% and 59% of MAs in SCP and DCP, respectively. OCTA can detect microvascular alterations in DM patients with no retinal lesions observed by dilated funduscopic examination [30]. However, vascular changes were only evaluated with fundus evaluation and OCTA; we did not perform fluorescein angiography to ensure the absence of MAs or other lesions. 

One of the limitations of our study is that we did not analyze the ICP. The images obtained with DRI-Triton OCT combine the ICP and the DCP. Another limitation is that we did not perform fluorescein angiography in the patient group, which could have detected more MAs than fundus examination. However, the main strength of our study lies in the inclusion of a cohort of very long-term DM1 patients. Combining imaging biomarkers with other predictive factors, such as genomics and metabolomics, along with clinical care, may potentially improve the prediction of the risk of developing DR. 

## 5. Conclusions

In conclusion, our study revealed a decrease in blood flow in all studied areas of the SCP and in all areas except the C area of the DCP in long-term DM1 patients without DR. Additionally, CC flow was found to be increased in the C area in the DM1 group compared to controls. Disease progression was associated with changes in the SCP FAZ area and horizontal and vertical diameters, with no modifications observed in the DCP FAZ. Furthermore, vascular abnormalities were detected in a significant proportion of patients. The flow in the SCP was mainly correlated with the duration of the disease and microalbuminuria levels. We will closely monitor these patients to observe the emergence of vascular signs in relation to our previous findings, with the aim of identifying biomarkers for DR development. Ultrawide-field OCTA and artificial intelligence could aid in establishing biomarkers for the early phenotypes of DR onset. 

## Figures and Tables

**Table 1:** Mean and standard deviation (SD) of metabolic parameters and disease duration in the DM1 and control groups. Abbreviations: HbA1c, glycosylated hemoglobin; HDL, high-density lipoprotein; LDL, low-density lipoprotein.

	DM1 Group (Mean ± SD)	Control Group (Mean ± SD)
Time of evolution (years)	26.56 ± 6.78	
Age at diagnosis (years)	19.10 ± 11.6	
HbA1c (%)	7.46 ± 0.90	
Glycemia (mg/dL)	159.51 ± 83.34	92.31 ± 15.74
Total cholesterol (mg/dL)	196.90 ± 31.89	197.50 ± 35.43
HDL cholesterol (mg/dL)	61.41 ± 14.43	58.02 ± 16.58
LDL cholesterol (mg/dL)	117.78 ± 27.37	120.87 ± 33.91
Urea (mg/dL)	35.72 ± 10.36	35.85 ± 12.16
Creatinine (mg/dL)	0.83 ± 0.19	0.85 ± 0.19
Albuminuria (mg/dL)	5.97 ± 7.22	

**Table 2:** Mean and standard deviation (SD) of the best corrected visual acuity (BCVA) using the LogMAR scale, spherical equivalent (SE) measured in diopters (D), axial length (AL) in mm, and intraocular pressure (IOP) in mmHg. No statistically significant differences were found between groups (*p* < 0.05).

	Control Group		DM1 Group		*p*
	Mean	SD	Mean	SD	
BCVA (LogMAR)	−0.08	±0.04	−0.04	±0.22	0.087
SE (D)	−0.76	±2.38	−0.97	±1.80	0.270
AL (mm)	23.78	±1.26	23.63	±1.04	0.383
IOP (mm Hg)	16.85	±2.47	16.59	±3.01	0.495

**Table 3:** Foveal avascular zone (FAZ) values in the superficial capillary plexus (SCP) and in the deep capillary plexus (DCP) in the control group and the DM1 group. The FAZ area is measured in µm^2^ and FAZ diameters in µm. Statistically significant values are shown in bold. Abbreviations: FAZ, foveal avascular zone; Ø, diameter.

	Control Group (*n* = 130)	DM1 Group (*n* = 78)	*p*
**SCP**			
FAZ area	255.34 ± 88.88	285.89 ± 100.67	**0.025**
FAZ Ø horizontal	577.02 ± 133.25	625.58 ± 141.59	**0.014**
FAZ Ø vertical	536.98 ± 119.72	572.58 ± 130.70	**0.048**
**DCP**			
FAZ area	195.63 ± 94.15	305.59 ± 109.49	0.492
FAZ Ø horizontal	674.85 ± 124.03	683.63 ± 139.67	0.641
FAZ Ø vertical	573.12 ± 103.20	563.32 ± 132.93	0.582

**Table 4:** Qualitative analysis of the alterations observed in the type 1 diabetes mellitus (DM1) group (%). Abbreviations: SCP, superficial capillary plexus; DCP, deep capillary plexus; CC, choriocapillaris.

	SCP	DCP	CC
FAZ abnormalities	53.8%	14.1%	
Capillary drop out	42.3%	48.7%	48.7%
Ischemia	46.1%	29.5%	17.9%
Microaneurysms	47.4%	56.4%	
Large microaneurysms	2.6%	2.6%	

**Table 5:** Significant correlations between the central (C) perfusion area of each plexus and FAZ parameters in the diabetic group. Correlation coefficient values (r) are shown in the first line of each row, and *p* values are shown in bold. Abbreviations: C, central; CC, choriocapillaris; DCP, deep capillary plexus; DM1, type 1 diabetes; FAZ, foveal avascular zone; HOR, horizontal; SCP, superficial capillary plexus; VER, vertical; Ø, diameter.

DM1 Group	SCP C	SCP FAZ	SCP FAZ Ø HOR	SCP FAZ Ø VER	DCP C	DCP FAZ	DCP FAZ Ø HOR	DCP FAZ Ø VER
SCP C	-	−0.748 ***p* < 0.001**	−0.772 ***p* < 0.001**	−0.737 ***p* < 0.001**	0.684 ***p* < 0.001**	−0.642 ***p* < 0.001**	−0.645 ***p* < 0.001**	−0.587 ***p* < 0.001**
DCP C	0.684 ***p* < 0.001**	−0.562 ***p* < 0.001**	−0.512 ***p* < 0.001**	−0.512 ***p* < 0.001**	-	−0.763 ***p* < 0.001**	−0.784 ***p* < 0.001**	−0.761 ***p* < 0.001**
CC C	-	-	-	-	-	-	-	-

## Data Availability

The data presented in this study are available upon reasonable request from the corresponding author due to patient privacy.
